# Essential Amino Acid-Enriched Diet Alleviates Dexamethasone-Induced Loss of Muscle Mass and Function through Stimulation of Myofibrillar Protein Synthesis and Improves Glucose Metabolism in Mice

**DOI:** 10.3390/metabo12010084

**Published:** 2022-01-16

**Authors:** Yeongmin Kim, Sanghee Park, Jinseok Lee, Jiwoong Jang, Jiyeon Jung, Jin-Ho Koh, Cheol Soo Choi, Robert R. Wolfe, Il-Young Kim

**Affiliations:** 1 Department of Health Sciences and Technology, GAIHST, Gachon University, Incheon 21999, Korea; 1893kin@gmail.com (Y.K.); jinseok0515@gmail.com (J.L.); jyjung031@gmail.com (J.J.); 2 Department of Molecular Medicine, College of Medicine, Gachon University, Incheon 21999, Korea; sangheepark1@gmail.com (S.P.); jinhokoh@yu.ac.kr (J.-H.K.); cschoi@gachon.ac.kr (C.S.C.); 3 Korea Mouse Metabolic Phenotyping Center, Lee Gil Ya Cancer and Diabetes Institute, Gachon University, Incheon 21999, Korea; korea81@hanmail.net; 4 Gil Medical Center, Department of Internal Medicine, Gachon University, Incheon 21565, Korea; 5 The Center for Translational Research in Aging and Longevity, Department of Geriatrics, Donald W. Reynolds Institute on Aging, University of Arkansas for Medical Sciences, Little Rock, AR 72205, USA; RWolfe2@uams.edu

**Keywords:** dexamethasone, muscle atrophy, essential amino acids, protein turnover, glucose metabolic flux

## Abstract

Dexamethasone (DEX) induces dysregulation of protein turnover, leading to muscle atrophy and impairment of glucose metabolism. Positive protein balance, i.e., rate of protein synthesis exceeding rate of protein degradation, can be induced by dietary essential amino acids (EAAs). In this study, we investigated the roles of an EAA-enriched diet in the regulation of muscle proteostasis and its impact on glucose metabolism in the DEX-induced muscle atrophy model. Mice were fed normal chow or EAA-enriched chow and were given daily injections of DEX over 10 days. We determined muscle mass and functions using treadmill running and ladder climbing exercises, protein kinetics using the D_2_O labeling method, molecular signaling using immunoblot analysis, and glucose metabolism using a U-^13^C_6_ glucose tracer during oral glucose tolerance test (OGTT). The EAA-enriched diet increased muscle mass, strength, and myofibrillar protein synthesis rate, concurrent with improved glucose metabolism (i.e., reduced plasma insulin concentrations and increased insulin sensitivity) during the OGTT. The U-^13^C_6_ glucose tracing revealed that the EAA-enriched diet increased glucose uptake and subsequent glycolytic flux. In sum, our results demonstrate a vital role for the EAA-enriched diet in alleviating the DEX-induced muscle atrophy through stimulation of myofibrillar proteins synthesis, which was associated with improved glucose metabolism.

## 1. Introduction

Muscle atrophy is defined as the loss of muscle mass and function (including strength and endurance) resulting from decreased protein synthesis and/or increased protein degradation [1,2]. Numerous physiological and pathological conditions, such as prolonged inactivity, cancer cachexia, aging, diabetes, and drug treatments, can cause muscle atrophy [3,4,5]. The various muscle atrophy conditions induced by dysregulation of muscle protein quality cause several unfavorable consequences, including a decrease in quality of life, an increase in mortality, and a dysregulation of the systemic energy homeostasis.

Dexamethasone (DEX) is a synthetic glucocorticoid, a commonly prescribed therapeutic compound for treating inflammatory, autoimmune, cancer, and neuromuscular disorders. Despite its pharmacological benefits in disease conditions, acute and prolonged treatment with DEX induces an imbalance in protein turnover and impaired glucose metabolism, leading to a decrease in both muscle mass and function. DEX treatment decreases muscle weight and inhibits the mTOR pathway, reduces the rate of muscle protein synthesis by 59%, and reduces muscle strength by up to 50% [6,7]. Moreover, DEX treatment is accompanied by insulin resistance in rodents [8,9]. In addition to the mTOR pathway, the ubiquitin-proteasome system (UPS) and autophagy are involved in maintaining protein quality control [10]. The activation of the UPS and autophagy is markedly increased under catabolic conditions, including DEX [11]. In particular, myofibrillar proteins are degraded by the enhanced UPS [12] and the expression of autophagy proteins (e.g., microtubule-associated protein II-light chain 3 (LC3-II), autophagy related 7 (ATG7)) have upregulated by DEX [13,14]. DEX can affect protein quality and quantity by suppressing protein synthesis and activating protein degradation. Furthermore, acute administration of DEX inhibits the metabolic clearance rate of glucose [15], and prolonged DEX treatment increases gluconeogenesis and decreases muscle glucose uptake [16]. Although DEX can be used to treat specific illnesses, an effective means against the DEX-mediated muscle atrophy and its negative impact on metabolism remains unresolved. Therefore, it is important to examine whether alterations occur both in muscle protein turnover and metabolism to better understand and in turn treat the DEX-induced muscle atrophy. However, previous studies have evaluated the effects of nutraceuticals, such as BCAA and omega-3 fatty acids, in vivo [17,18] without examinations on both protein turnover and metabolism.

Amino acids act as building blocks for proteins and produce favorable effects alone or together on health and diseases [19]. Particularly, essential amino acids (EAAs) are the main drivers that induce positive muscle protein balance (i.e., gains in muscle mass) [20,21,22,23] and improve muscle function via affecting various factors, including protein metabolism, mitochondrial biogenesis, and energy metabolism. In humans, it was shown that EAA supplementation for 1 week led to a robust anabolic stimulation in skeletal muscle and enhanced recovery capacity through protecting myofibrillar protein degradation following exercise [24,25]. Additionally, EAAs promote mitochondrial biogenesis and muscle function as well as suppress plasma glucocorticoid concentration [21,26,27]. Furthermore, some EAAs have been demonstrated to stimulate glucose uptake in rodents [28,29,30] during OGTT. Therefore, EAA supplementation has been utilized as a potential nutritional treatment for various muscle atrophy conditions, including aging [31], obesity [32], and osteoporosis [33]. According to the above-mentioned results, it is suggested that EAAs may alleviate muscle atrophy induced by DEX via enhancement of muscle protein synthesis and glucose metabolism. However, no studies have been conducted regarding the potential positive impact on the DEX-mediated dysregulation of muscle protein balance and glucose metabolism.

Therefore, in the present study, we tested hypotheses that a diet rich in EAAs will alleviate muscle atrophy and reverse impaired glucose metabolism induced by dexamethasone treatment. Here we show that an EAA-enriched diet protects muscle from the DEX-induced atrophy via enhancement of muscle protein synthesis and insulin stimulated-glucose metabolism.

## 2. Results

### 2.1. The EAA-Enriched Diet Alleviates Loss of Muscle Mass and Strength in Muscle Atrophy by DEX

**Figure 1:**
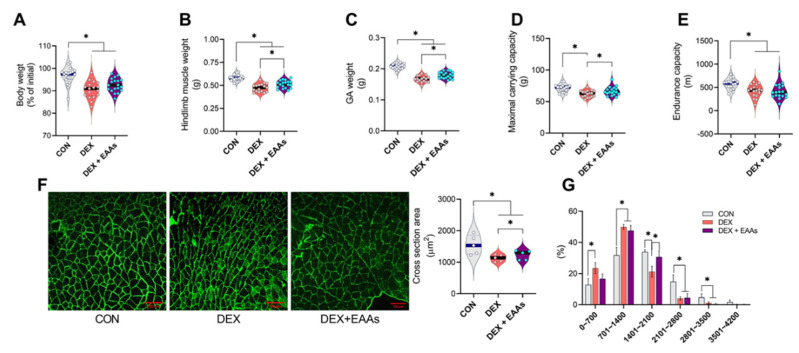
The EAA-enriched diet alleviated the reduction of muscle mass and strength on DEX-induced muscle atrophy. (**A**) Changes in body weight after 10 days of injection and/or the EAAs diet (*n* = 17–19). (**B**) Total hindlimb and (**C**) GA muscle weight (*n* = 17–19). (**D**) Maximal carrying capacity (*n* = 17–19). (**E**) Endurance capacity (*n* = 17–19). (**F**) Cross-section area in GA muscle (*n* = 5). (**G**) Muscle fiber frequency distribution of GA muscle (*n* = 5). Data are presented as mean ± S.E. * Significant difference between labeled groups (* *p* < 0.05). CON, control; DEX, dexamethasone; EAAs, essential amino acids; GA, gastrocnemius.

Although DEX is widely used as a treatment for various disease conditions, muscle atrophy is a major side effect of DEX. In contrast, an EAA-enriched diet is known to induce an anabolic response [23,34]. Thus, we examined whether an EAA-enriched diet can prevent the loss of muscle and function induced by DEX. We found that 20 mg/kg DEX daily injection for 10 days significantly decreased body weight in all groups (Figure 1A). The EAA-enriched diet improved muscle mass of total hindlimb (gastrocnemius, soleus, plantaris, extensor digitorum longus, tibialis anterior) and gastrocnemius (GA) in DEX-treated mice (Figure 1B,C). It is well known that an EAA-enriched diet induces not only net muscle protein synthesis (i.e., gains in muscle mass) but also improvement of muscle function. Consistent with changes in muscle mass, we found a significant reduction in cross-sectional area (CSA) of GA muscle by DEX that was partially prevented by the EAA-enriched diet (Figure 1F,G). These results suggest that the EAA-enriched diet can prevent decreases in muscle mass and strength induced by DEX via attenuating decreases in middle-sized fibers. Similarly, we found that DEX decreased the maximal carrying capacity (MCC), which was prevented by EAA-enriched diet (Figure 1D). Contrary to our expectation based on the previous study [26,35], we found that the EAA-enriched diet did not improve endurance capacity or mitochondrial biogenesis (Figure 1E).

### 2.2. The EAA-Enriched Diet Increased Myofibrillar Protein Synthesis in DEX Treated Mice

**Figure 2:**
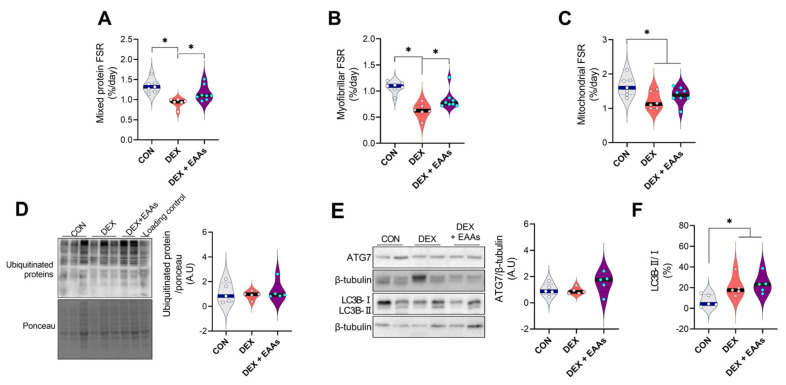
The EAA-enriched diet promotes myofibrillar protein synthesis rate but not the protein degradation pathway by DEX treatment in GA muscle. (**A**) Rates of integrated mixed muscle protein synthesis rate (*n* = 6–8). (**B**) Rates of myofibrillar protein synthesis rate (*n* = 6–8). (**C**) Mitochondrial protein synthesis rate (*n* = 6–8). (**D**) Relative protein expression levels of ubiquitinated proteins (expression levels were normalized by total proteins, *n* = 5). (**E**) Relative protein expression levels of ATG7 and (**F**) the ratio of LC3B-II/I (*n* = 5). * Significant difference between labeled groups (* *p* < 0.05). CON, control; DEX, dexamethasone; EAAs, essential amino acids; GA, gastrocnemius; FSR, fractional protein synthetic rate; ATG7, autophagy related7; LC3B, microtubule-associated protein II-light chain 3.

Muscle mass is determined by the balance between rates of protein synthesis and degradation. DEX induces muscle atrophy through the imbalance in protein metabolism resulting from inhibition of protein synthesis rate and/or activation of protein degradation pathways, which are promoted by dysregulation of UPS and autophagy pathway [36,37]. Since the EAA-enriched diet has been shown to increase muscle mass through enhanced muscle protein synthesis rate and decreased protein degradation in normal conditions [38,39], we tested whether the EAA-enriched diet could increase protein synthesis even in a condition in which DEX is continuously injected in mice. As expected, we found that DEX significantly decreased integrated mixed muscle fractional protein synthetic rate (FSR) in GA muscle, and this decrease was prevented by the EAA-enriched diet (Figure 2A). However, Akt/mTORC1 pathway has not differed in all groups (Figure S1A–C). Interestingly, the EAA-enriched diet prevented a decrease in FSR of myofibrillar proteins (Figure 2B). Furthermore, we found that the integrated mixed muscle FSR and myofibrillar FSR of the EAA-enriched diet were not significantly different from CON. However, mitochondrial proteins FSR have not been differed between DEX and EAA-enriched diet (Figure 2C). These results are in accordance with the results on the physical performance tests: we found an improvement in MCC but not in endurance capacity when treated with the EAA-enriched diet (Figure 1C,D). Since UPS and autophagy are major aspects of control of protein breakdown, we identified the ubiquitination of proteins, ATG7 and LC3B. We found that DEX and/or the EAA-enriched diet have not changed the amounts of UPS and ATG7 protein abundance compared to CON (Figure 2D,E). DEX increased the ratio of LC3B-II/I than CON, however, the EAA-enriched diet did not change the ratio of LC3B-II/I protein amount than DEX (Figure 2F). Overall, these data indicate that the EAA-enriched diet protects skeletal muscle atrophy via enhanced muscle protein synthesis rate, (mainly myofibrillar protein synthesis), without changing the protein degradation pathways that DEX promotes.

### 2.3. The EAA-Enriched Diet Improves Insulin Sensitivity and Glucose Uptake in the Presence of DEX Treatment

**Figure 3:**
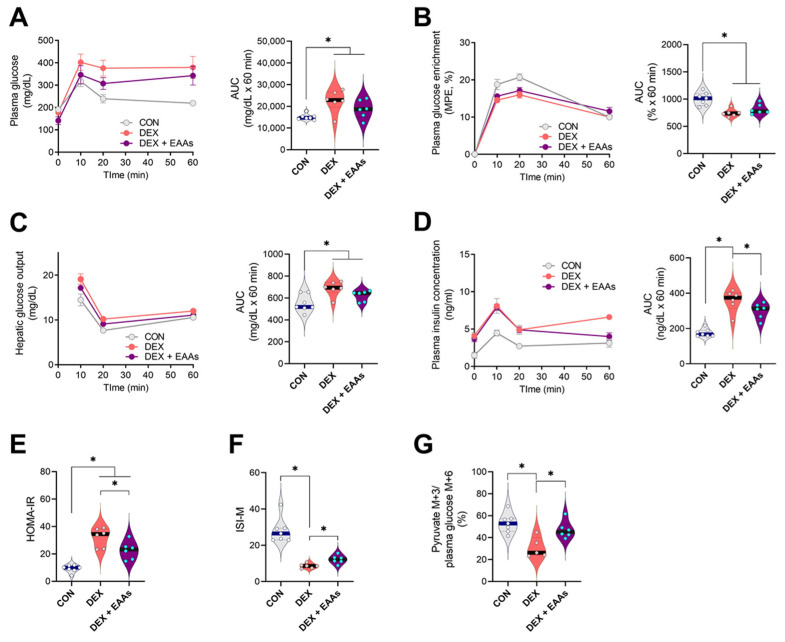
The EAA-enriched diet improved impaired insulin sensitivity and glucose uptake in DEX-induced muscle atrophy. (**A**) Plasma glucose concentration during the oral glucose tolerance test (OGTT) (*n* = 6–7). (**B**) Plasma glucose enrichment during the OGTT (*n* = 6–7). (**C**) Hepatic glucose output during the OGTT (*n* = 6–7). (**D**) Plasma insulin concentrations during the OGTT (*n* = 6–7). (**E**) HOMA-IR (*n* = 6–7). (**F**) Matsuda insulin sensitivity index (*n* = 6–7). (**G**) GA muscle pyruvate M+3 to plasma glucose M+6 ratio (*n* = 6–7). Data are presented as mean ± S.E. * Significant difference between labeled groups (* *p* < 0.05). CON, control; DEX, dexamethasone; EAAs, essential amino acids; GA, gastrocnemius, MPE, mole percent excess; AUC; area under curve; HOMA-IR, homeostatic model assessment of insulin resistance; ISI-M, Matsuda-DeFronzo insulin sensitivity index.

DEX can activate gluconeogenesis and reduce glucose uptake in muscle by inducing insulin resistance [40,41]. DEX could impair regulation of glucose metabolism as a consequence of muscle atrophy, which can be reversed by increased dietary EAAs [27,28]. Thus, we examined the effect of the EAA-enriched diet on glucose metabolism in the presence of DEX using a glucose stable isotope tracer during an oral glucose tolerance test (OGTT). Plasma glucose concentration is regulated by rates of hepatic glucose output and glucose uptake [42]. We first determined that DEX lowered the enrichment of glucose after the OGTT (Figure 3B), reflecting a reduced suppression of hepatic glucose output as compared to CON, and that the EAA-enriched diet did not affect this response (Figure 3C). Interestingly, while DEX increased plasma insulin concentrations and worsened insulin resistance in fasting and postprandial states (HOMA-IR index and ISI-M) compared to CON, the decrease in insulin sensitivity was partially revered by the EAA-enriched diet (Figure 3D–F). We also analyzed pyruvate enrichment (normalized by plasma glucose enrichment) in the GA muscle, which reflected glucose uptake and subsequent glycolytic flux. We found that DEX decreased pyruvate enrichment in the GA muscle compared to CON, which was prevented by the EAA-enriched diet (Figure 3G). Collectively, these findings demonstrated that the EAA-enriched diet prevents the DEX-induced impaired insulin sensitivity and muscle glucose uptake.

## 3. Discussion

The current study provides experimental evidence that the EAA-enriched diet alleviates the DEX-induced muscle atrophy by stimulating myofibrillar protein synthesis, and also improves glucose metabolism. The improved insulin sensitivity in the animals receiving the EAA-enriched diet may have been linked to the increased muscle protein synthesis, which minimized the extent of muscle atrophy resulting from DEX. Elucidation of the favorable effect of the EAA-enriched diet on the DEX-induced muscle atrophy model extends potential opportunities for future studies of muscle wasting in clinical conditions that are induced by adverse side effects of drugs.

DEX is used to treat many conditions related to immune function, cancer, and neuromuscular disorders [43,44]. However, chronic treatment with DEX decreases muscle mass and function [45]. Thus, we found that DEX decreased GA muscle mass by approximately 20% (Figure 1B–E). In addition, maximal carrying capacity was reduced by 16%, and endurance capacity was reduced by 26%. It is well known that a diet rich in EAAs induces gains in muscle net protein balance (i.e., gains in muscle mass) in normal [21,22] and pathological conditions [20]. Therefore, we performed the experiment to test whether an EAA-enriched diet could protect the muscle against DEX-induced muscle atrophy. As expected, we found that the EAA-enriched diet improved muscle mass by 5% and strength by 10% even in the condition in which DEX was continuously stimulating muscle atrophy. However, the EAA-enriched diet did not improve endurance capacity and mitochondrial biogenesis (Figure 1E and Figure 2C). Maybe this difference of result has been partly related to differences in the format of the EAA provision. Because of that, it will be a topic of future study (EAA supplement vs. EAA-enriched diet). Importantly, we found that the EAA-enriched diet improved myofibrillar protein synthesis rate that was decreased by DEX treatment without changes in Akt/mTORC1 signaling pathway (Figure S1). It is not uncommon that disparity between rate of muscle protein synthesis and Akt/mTORC1 signaling pathway exists as the signaling is one of many factors, including the availability of EAAs that determine the rate of muscle protein synthesis [46]. In this study, it is also possible that mTORC1 independent processes may regulate protein synthesis [47]. Myofibrillar proteins (actin, myosin, and other muscle contraction-related proteins) are involved in the contraction and compose 50–55% of the total muscle protein [48]. We also anticipated that the EAA-enriched diet would improve endurance capacity, as it has been demonstrated that EAAs may prevent mitochondrial dysfunction and oxidative stress in muscle atrophy conditions [49]. However, we did not observe an enhancement of endurance capacity and mitochondrial protein synthesis rate by the EAA-enriched diet.

At the molecular level, muscle protein mass is regulated by rates of protein synthesis and degradation [50]. DEX has been shown to trigger muscle atrophy by enhanced protein degradation machinery, i.e., UPS and autophagy [35,51,52]. However, despite the promotion of muscle atrophy by DEX in our study, we could not find changes in the amount of ubiquitin protein. This disparity might be due in part to differences in rodent species and tissue sampling times as well as other unknown factors. However, another muscle atrophy mechanism, i.e., autophagy, was increased by DEX as demonstrated by an increase in the ratio of LC3B-II/I [37]. It is an interesting finding that the EAA-enriched diet prevented net muscle protein degradation induced by DEX despite the absence of any change in autophagy, which is believed to be a main driver of the DEX-mediated muscle atrophy [14]. Previous studies have shown that the autophagy pathway can be enhanced to regulate protein quality through facilitating degradation of the aggregated pathogenic protein [53,54]. Thus, an increase in autophagy mechanism may be good or bad, namely, acting as a double-edged sword depending on conditions. However, the effect of the EAA-enriched diet on autophagy in relation to muscle mass and function remains unclear. Thus, further studies are required to find roles placed by autophagy (changes) in the enhancement of muscle function with the EAA-enriched diet.

Skeletal muscle accounts for approximately 80% of the insulin-stimulated glucose uptake [55]. Whole-body glucose metabolism is regulated by the balance between the rate of glucose appearance and the rate of glucose disappearance [56,57,58]. In the normal fed condition, insulin inhibits hepatic glucose output and activates glucose uptake in the peripheral tissues, mainly in the skeletal muscle [59,60]. It is known that DEX induces insulin resistance and contributes to elevated hepatic glucose output and decreased muscle glucose uptake [61]. Thus, it is likely that DEX-induced muscle atrophy plays a role in the progression of metabolic diseases, such as insulin resistance and type 2 diabetes mellitus. In fact, since insulin resistance and dysregulation of glucose uptake can directly induce muscle atrophy [62,63], muscle mass and metabolic function are linked. In this context, it is possible that the improved glucose metabolism in the DEX animals fed the EAA-enriched diet was due to the concurrent increase in muscle mass. However, a further studies exploring mechanisms by which the EAA-diet induces improved insulin sensitivity and muscle glucose uptake are required. Furthermore, it is possible that alternative ways of providing EAAs, such as consumption of EAAs alone between meals, would have different metabolic effects. Lastly, it is important to confirm the results from the present study in clinical outcome trials.

## 4. Materials and Methods

### 4.1. Animals Models and Treatments

C57BL/6 mice at the age of 8 weeks were used for all experiments. The mice were randomly divided into three experimental groups: control (CON, *n* = 19), dexamethasone treat + normal chow (DEX, *n* = 17), and dexamethasone treat + EAAs chow (DEX + EAAs, *n* = 19). The same experiment was repeated three times for FSR (*n* = 6–8), OGTT (*n* = 6–7), and molecular signaling pathway (*n* = 5) analysis, and mice were distributed according to each experiment. The mice were maintained at 22 ± 1 °C on a 12-h light/dark cycle, with ad libitum food and water. Normal groups consumed a normal chow (D10001, open-source diets, New Brunswick, NJ, USA) consisting of 21% Kcal from protein, 68% kcal carbohydrate, and 12% kcal fat. To induce increases of EAA intake, EAA-enriched diet groups were fed chow containing twice the EAAs (Table 1, A19071101, open-source diets, New Brunswick, NJ, USA) as normal chow protein. Normal and EAA-enriched chow were matched for total kcal. The body weight was measured at the same time every day. To generate a muscle atrophy model, we injected 20 mg/kg/day dexamethasone (D4902, Sigma-Aldrich, St Louis, MO, USA) in an intraperitoneal manner for 10 days. All mice experiments were approved by the Lee Gil Ya Cancer and Diabetes Institute. 

### 4.2. Muscle Function Tests

Before the maximal carrying capacity test, familiarization exercise sessions without load for 3 days were conducted using a 1-m ladder with rungs that were 15 mm apart and inclined at 85°. During the test, the mice climbed the ladder with 50%, 70%, 90%, and 100% of the body weight, and the load was added by 3 g until the test was completed. The mice rested for 2 min at the top of the ladder apparatus [22]. The sum of the highest load successfully carried and bodyweight was considered as the maximum carrying capacity.

For the exhaustion test, mice were adapted on a treadmill for 2 days before the test. The adaptation involved mice running for 30 min at 8 m/min. At the start of the exhaustion test, mice started running at the speed of 8 m/min. The running speed was increased at 1 m/min every 2 min until mice were exhausted. The criterion of exhaustion was defined as the point at which mice stayed >5 s on the electric shocker without trying to resume running even if pushed [22].

### 4.3. Immunofluorescence Staining

To measure muscle cross-section area, muscle sections were incubated overnight at 4 °C with 1:500 dilution of rabbit anti-laminin (#L9393, Sigma-Aldrich, St Louis, MO, USA). After washes in PBS, muscle sections were incubated for 1 h with 1:2000 dilution of Alexa Fluor 488-conjugated anti-rabbit IgG (#A-21121, Invitrogen, Waltham, MA, USA). Images were obtained using a confocal microscope (Zeiss LSM 700, Oberkochen, Germany) at the same light setting, exposure time, and magnification. For fiber CSA calculation, ImageJ image analysis software (National Institutes of Health) was used.

### 4.4. Oral Glucose Tolerance Test

The mice were fasted for 6 h. At the start of the oral glucose tolerance test, the body weight was measured, and a baseline plasma was collected via the tail vein for the determination of fasting glucose and insulin prior to an oral gavage of a 1.5 g/kg body weight glucose (1:1 ratio of [U-^13^C_6_]glucose and unlabeled glucose). Plasma was collected at 0, 10, 20, and 60 min post-gavage using capillary tubes. Plasma glucose concentration was measured immediately using a glucometer (GM9, Analox Instruments, Stourbridge, UK). Plasma insulin was determined using an insulin ELISA kit (80-INSMSU-E10, ALPCO, Salem, MA, USA). The homeostasis model assessment IR (HOMA-IR) index and Matsuda insulin sensitivity index were calculated as described in the previous study [64]. The area under the curve (AUC) was calculated for each adjacent time point of plasma glucose concentration, enrichment, hepatic glucose output, and insulin concentration.

### 4.5. The Isolation of Myofibrillar and Mitochondrial Related Proteins

Differential centrifugation method used to extract myofibrillar and mitochondrial subtractions as previously describe [65]. Briefly, GA muscle was homogenized in a buffer consisting of 550 mM KCl, 5 mM EGTA and 100 mM MOPS and centrifuged at 800× *g* for 5 min at 4 °C for precipitating myofibrillar fraction. The supernatant was transferred and centrifuged further at 7000× *g* for 10 min at 4 °C for collecting mitochondrial fraction.

### 4.6. D_2_O Labeling and Protein Synthetic Rate Calculation

All mice received an intraperitoneal injection of 35 μL/g body weight of 99%, D_2_O (DLM-4, Cambridge isotope laboratories, Tewksbury, MA, USA) with 0.9% NaCl at 0 day. To maintain the D_2_O enrichment in plasma, the mice received drinking water with 8% D_2_O enrichment for 10 days. Calculations of muscle protein fractional synthesis rate were determined, based on the precursor-produce relation [57,58].

FSR (%/t) = (MPE_Ala_)/(MPE_BW_ × 3.7) × 100

*k*_s_ = −ln(1 − FSR)/*t*

where FSR is the fractional synthetic rate of muscle, MPE (mole percent excess) is calculated as a tracer to tracee ratio (TTR)/(TTR+1), and MPE_Ala_ represents the total ^2^H-labeling of protein bound alanine. “3.7” represents the potential exchange site of ^2^H alanine from the body fluid, MPE_BW_ represents the labeling of body fluid and “*t*” is the labeling period (time). *k*_s_ is the fractional synthesis rate constant [66].

### 4.7. Stable Isotope Enrichment Analysis

Body fluid and muscle protein enrichment were analyzed as previously described [21,67]. Briefly, the acetone exchange method was used to measure ^2^H enrichment in plasma. For the analysis of alanine enrichment, muscle tissue was homogenized with 6% perchloric acid, precipitated at 21,000× *g* at 4 °C and hydrolyzed into amino acids at 100 °C for 16 h. The free amino acids from the hydrolyzed sample were extracted via cation-exchange resin columns and dried under Speed vac (Savant Instruments, Farmingdale, NY). To investigate glycolytic pathway and TCA cycle metabolic flux in skeletal muscle, muscle tissues were quickly dissected and immediately frozen in liquid nitrogen. GA muscle was homogenized with 70% ACN and 30% distilled water and centrifuged at 14,000× *g* for 5 min at 4 °C. After centrifuging, the supernatant was transferred to a new tube. The supernatant was dried using Speed Vac. Derivatization for GC-MS was initiated in 50 µL of 2% MOX in pyridine for 90 min at 37 °C. Then, 50 µL of MTBSFTA + 1% TBDMSCIS (#00942, Sigma-Aldrich, St Louis, MO, USA) was added and incubated for 30 min at 60 °C. Lastly, the samples were centrifuged at 14,000× *g* for 5 min at room temperature and 75 µL of supernatant was transferred to a GC-MS vial.

### 4.8. Immunoblot Analysis

Immunoblotting was performed as described previously [22]. In brief, GA muscle was denatured in lysis buffer and protein concentration was measured using a BCA assay kit (#A53225, Thermo Fisher Scientific, Waltham, MA, USA). A total protein concentration of 20 µg was resolved by SDS-PAGE and transferred to polyvinylidene fluoride (PVDF) membranes. Primary antibodies, including AKT (#9272), p-AKT (Ser473)(#9271), mTOR (#4844S), p-mTOR (Ser2448)(#2971), rpS6 (#2217), p-rpS6 (Ser235/236)(#4856S), ATG7 (#8558), LC3B (#NB100-2220, Novus Biologicals, CO, USA), ubiquitin (#3933S, Cell Signaling Technology, Danvers, MA, USA), and β-tubulin (#05-661, Merck Millipore, Burlington, MA, USA) were diluted to a 1:1000 concentration ratio and incubated overnight at 4 °C. After an hour of incubation secondary antibody, bands were detected using ECL solution and quantified with ImageJ image analysis software (National Institutes of Health).

### 4.9. Statistical Analysis

The data were expressed as mean ± S.E and analyzed using GraphPad Prism 9 and SPSS for window version 21.0. Data were analyzed to determine the main effects of interventions groups on variables by one-way ANOVA (followed by Fisher’s least significant difference test). The area under curve (AUC) for the blood glucose concentration, enrichment, insulin concentration during the OGTT was determined using the trapezoidal method. Statistical significance was set at *p* < 0.05.

## 5. Conclusions

We have evaluated the effect of an EAA-enriched diet on the DEX-induced muscle loss and function and its impact on glucose metabolism. The EAA-enriched diet protected from the DEX-induced muscle atrophy via enhanced rates of protein synthesis and insulin stimulated-glucose metabolism. These findings suggest a nutraceutical potential for an EAA-enriched diet that may counteract DEX-induced muscle atrophy in clinical settings.

## Figures and Tables

**Table 1:** EAA compositions of normal chow and EAA-enriched chow.

Amino Acids (g/100 g)	Normal Chow (D10001, in Protein)	EAAs Chow (A19071101)
L-Isoleucine	0.27	0.8
L-Leucine	0.53	1.6
L-Lysine	0.43	1.3
L-Methionine	0.27	0.8
L-Phenylalanine	0.27	0.8
L-Threonine	0.23	0.7
L-Tryptophan	0.07	0.2
L-Valine	0.3	0.9
L-Histidine	0.17	0.5
Total EAAs	2.54	7.6

## Data Availability

The data presented in this study are available in article and supplementary material.

## Supplementary Materials

The following are available online at https://www.mdpi.com/article/10.3390/metabo12010084/s1. Figure S1: Ratio of phosphorylated Akt/mTORC1 related proteins.
