# Supplementary material for: Essential Amino Acid-Enriched Diet Alleviates Dexamethasone-Induced Loss of Muscle Mass and Function through Stimulation of Myofibrillar Protein Synthesis and Improves Glucose Metabolism in Mice
Source: Metabolites. 2022 Jan 16;12(1):84. doi: 10.3390/metabo12010084 (PMC8778336; doi:10.3390/metabo12010084)
Supplement: Supplementary file 1 [file metabolites-12-00084-s001.zip › metabolites-1555916-supplementary.pdf]

## Supplementary data

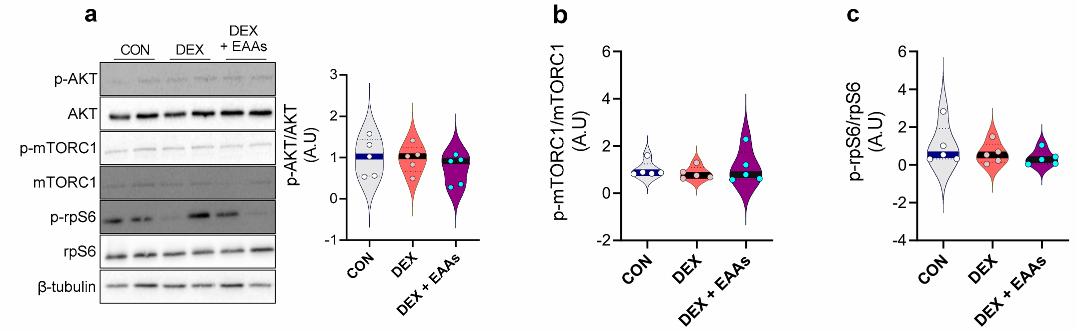


**Supplementary Figure S1.** Ratio of phosphorylated (**a**) Akt, (**b**) mTORC1, and (**c**) rpS6 proteins (normalized by each total protein expression). Akt, A protein cloned from the v-akt oncogene of retrovirus AKT8; mTORC1, Mammalian target of rapamycin complex1; rpS6, Ribosomal protein S6.
